# Patterns of periodic holes created by increased cell motility

**DOI:** 10.1098/rsfs.2012.0001

**Published:** 2012-03-28

**Authors:** Ting-Hsuan Chen, Chunyan Guo, Xin Zhao, Yucheng Yao, Kristina I. Boström, Margaret N. Wong, Yin Tintut, Linda L. Demer, Chih-Ming Ho, Alan Garfinkel

**Affiliations:** 1 Mechanical and Aerospace Engineering Department, University of California, Los Angeles, CA, USA; 2 Department of Bioengineering, University of California, Los Angeles, CA, USA; 3 Department of Medicine, University of California, Los Angeles, CA, USA; 4 Department of Integrative Biology and Physiology, University of California, Los Angeles, CA, USA; 5 Molecular Biology Institute, University of California, Los Angeles, CA, USA; 6 Institute of Robotics and Automatic Information Systems, Nankai University, Tianjin, Peoples's Republic of China

**Keywords:** pattern formation, cell motility, adult stem cells, self-organization, Turing instability

## Abstract

The reaction and diffusion of morphogens is a mechanism widely used to explain many spatial patterns in physics, chemistry and developmental biology. However, because experimental control is limited in most biological systems, it is often unclear what mechanisms account for the biological patterns that arise. Here, we study a biological model of cultured vascular mesenchymal cells (VMCs), which normally self-organize into aggregates that form into labyrinthine configurations. We use an experimental control and a mathematical model that includes reacting and diffusing morphogens and a third variable reflecting local cell density. With direct measurements showing that cell motility was increased ninefold and threefold by inhibiting either Rho kinase or non-muscle myosin-II, respectively, our experimental results and mathematical modelling demonstrate that increased motility alters the multicellular pattern of the VMC cultures, from labyrinthine to a pattern of periodic holes. These results suggest implications for the tissue engineering of functional replacements for trabecular or spongy tissue such as endocardium and bone.

## 1. Introduction

Morphogenesis consists of local activities that contribute collaboratively to global pattern formation. In the paradigm introduced by Turing [1], these patterns arise as solutions to partial differential equations (PDEs) governing the local reaction and diffusion of chemical ‘morphogens’. Instabilities of the homogeneous spatial equilibrium create periodic patterns of ‘spots’, ‘stripes’, ‘labyrinths’ and ‘holes’, which have been studied theoretically and in a number of physical, chemical and biological systems [2–4]. In particular, patterns of periodic holes have been observed in systems as diverse as arid spots in vegetation and in the pigmentation of tropical fish [5–8]. In physiology, hole patterns can serve as a model for the structure of trabecular or spongy tissue, such as bone. We are particularly interested in how changes in cell physiology, such as enhanced cell migration, get integrated and amplified into the development of tissue architecture. It is often difficult to study these questions because the experimental control of parameters required to explore such mechanisms is generally not available in observational studies of biological systems.

Here, we use cultured vascular mesenchymal cells (VMCs), stem cell-like multipotent cells that differentiate and self-organize into aggregates in labyrinthine and spot configurations by a Turing-like instability [9]. In these cultures, we are able to control and measure the parameters that govern cell motility. We found that experimental increases in ‘random’ cell motility, whether by inhibition of Rho kinase (ROCK) or of non-muscle myosin-II (NMM-II), changed the pattern formation of these cultures from a labyrinthine pattern to a novel observation, a pattern of periodic holes. (The term ‘random motility’ is used in the literature to distinguish this kind of motility, which has no directional preference, from chemotactic motility, which is always in the preferred direction of chemical morphogen gradients.)

The experimental results are consistent with mathematical predictions from computational simulations reflecting realistic morphogen and cellular kinetics. Thus, with direct evidence showing how cellular activities interplay with the reaction and diffusion of chemical morphogens, our findings attempt to bridge the gap between cell physiology and tissue-level morphogenesis.

## 2. Material and methods

### 2.1. Cell culture

VMCs were isolated as described [10] and cultured in Dulbecco's modified Eagle medium supplemented with 15 per cent heat-inactivated foetal bovine serum and 1 per cent penicillin/streptomycin (10 000 IU/10 000 µg ml^−1^; all from Invitrogen, CA, USA). Cells were incubated at 37°C in a humidified incubator (5% CO_2_ and 95% air) and passaged every 3 days. For multicellular pattern formation, cells were plated on plastic substrate (200 000 cells on 35 mm plastic dishes) with media changes every 3 days. For ROCK and NMM-II inhibition, Y27632 (10 μM, Ascent Scientific, NJ, USA) and blebbistatin (10 μM, Sigma–Aldrich, St Louis, MO, USA) were added at day 0 and replenished with each media change. After 10–14 days, cultures were stained with haematoxylin for 15 min to reveal multicellular aggregates.

### 2.2. Reaction kinetics experiments

Bovine aortic endothelial cells were cultured and transfected as previously described [11]. We experimentally varied concentrations of bone morphogenetic protein (BMP) and matrix gamma-carboxyglutamic acid protein (MGP) presented to the cells: the BMP treatments used recombinant human BMP-4 (R&D Systems), and the MGP challenges used an N-terminally FLAG-tagged human MGP (hMGP) vector, constructed as previously described [11], to express MGP.

The cellular response to BMP treatments (over 24 h) was recorded using luciferase assays, where the BMP-responsive luciferase reporter gene was transfected into the cells, and luciferase assays were performed as described previously and normalized to Renilla luciferase [11].

The MGP response was measured by gene expression. Cells were plated onto 24-well plates at 3 × 10^4^ cells per well 20–24 h prior to transfection [12]. RNA was collected 24 h after the start of the BMP-4 treatment or transfection with the hMGP expression vector. Real-time PCR analysis was performed as previously described, and glyceraldehyde 3-phosphate dehydrogenase was used as control gene [12,13]. Primers and probes specific for bovine MGP were obtained from Applied Biosystems as part of TaqMan Gene Expression Assays.

### 2.3. Shadow mask plating

**Figure 1.**
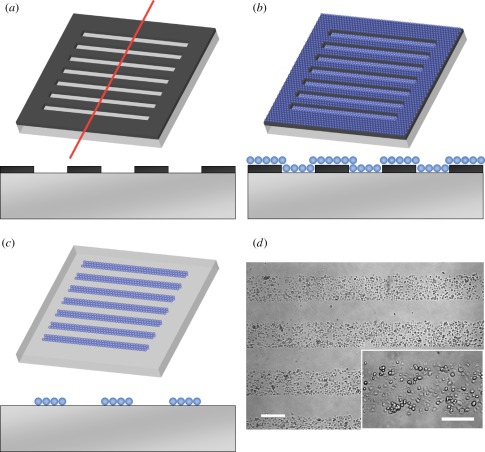
Procedure of shadow mask plating. (*a*) The mask is placed on the culture dish uniformly coated with fibronectin (upper figure). The lower figure is a schematic representing the side view along a line corresponding to the red line above. (*b*) Cells are plated through the mask allowing only the cells within the windows to adhere to the fibronectin. (*c*) Alternating stripes of cells and cell-free regions after removal of the mask. (*d*) Microscopic images after shadow mask plating show rectangular stripes of cells. Scale bar, 300 µm and 200 µm (inset).

The mask was made of stainless steel (2 cm × 2 cm × 100 μm, NW Etch, WA, USA) containing 25 parallel windows (300 μm × 1.5 cm) spaced 300 μm apart [14]. Prior to plating, the tissue culture dish was first uniformly coated with fibronectin solution (50 μg ml^−1^) at 4°C for 15 min. After brief washings, the mask was overlaid on the fibronectin substrate with a permanent magnet attached underneath the culture dish to immobilize the mask (figure 1*a*). Thereafter, cells were plated through the mask for 30 min (200 000 cells in 500 μl) followed by removal of the shadow mask (figure 1*b,c*), leaving alternating 300-μm-wide stripes of cells and cell-free regions (figure 1*d*). For inhibition of ROCK or NMM-II, the inhibitors were added at plating and presented during the entire culture.

### 2.4. Immunofluorescent staining

To visualize the expansion of cell stripes, cells were fixed in cold methanol (−20°C) for 10 min, 6 h after plating, blocked with Image-iT FX signal enhancer (Invitrogen) at room temperature for 30 min, and labelled with NMM-IIa antibody (1 : 1000, Covance, CA, USA) at room temperature for 1 h. The NMM-IIa antibodies were subsequently labelled with secondary antibodies for 30 min (Alexa Fluor 555 anti-rabbit IgG antibodies, 1 : 500; Invitrogen), and mounted by ProLong GOLD antifade with DAPI (Invitrogen). The images were acquired using a charge-coupled device (Coolsnap ES, Photometrics) equipped with an inverted microscope (Eclipse ECLIPSE TE2000, Nikon) with excitation wavelengths of 510–560 nm.

### 2.5. Automated image processing

**Figure 2.**
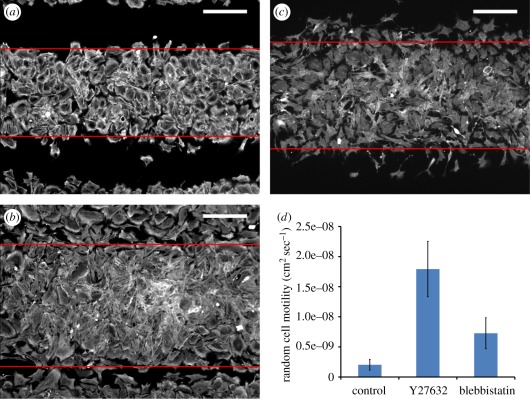
Determination of random cell motility using a wound-healing cell migration assay. (*a*–*c*) The expansion of cell fronts cultured in (*a*) normal condition, or with (*b*) ROCK inhibition or (*c*) NMM-II inhibition, where the edges (red lines) were identified through automated image processing. Scale bar, 200 µm. (*d*) The measured random cell motility (mean ± s.d.) in different culture conditions.

Images were automatically processed using image segmentation, a labelling algorithm and an edge-detection method. Specifically, using an intensity histogram from a grey-scale image, the image was segmented by selecting the midpoint between the representative values for ‘background’ and ‘cells’. Next, a labelling algorithm using a ‘bwlabel’ function in Matlab (Natick, MA, USA) was applied to locate each connected bright region so that the cell front can be identified by excluding small closed-loop regions. Subsequently, the edges of the cell front were determined by a threshold that defines a specific number of bright pixels appearing in each row of the segmented image (red lines in figure 2*a–c*). Then, successive wavefronts were used to calculate the front speed *V*, obtained by calculating the speed of expansion of the stripe width over 6 h. This speed was then used in Fisher's equation to estimate the cellular diffusion coefficient.

### 2.6. Mathematical model

Turing's original model postulated two chemical morphogens reacting and diffusing. Our experimental intervention, changing *cell* motility, cannot be directly modelled in the original Turing model, which is a two-variable PDE in the concentrations of two chemical morphogens. Instead, the model requires a third variable to reflect cell density and the cellular processes of chemotaxis and random cell motility. Such models are well known; following the work of Keller & Segel [15] and others [16–18], we modelled our system as the reaction and diffusion of a slowly diffusing chemical activator *a*, and its rapidly diffusing inhibitor *h*, considered as functions over a two-dimensional spatial domain (*x*, *y*). Our reaction terms used a version of Gierer–Meinhardt kinetics [18]. The third variable *N*(*x*, *y*) represents local cell density.2.1
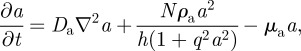
2.2
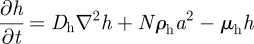
2.3
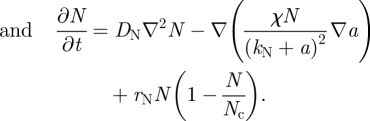


The first two equations were described previously [9]. Briefly, in equation (2.1), the production of activator follows autocatalytic reaction kinetics and is also regulated by the inhibitor and by its own decay (at a rate *μ*_a_). For activation, we use a sigmoidal form *ρ*_a_*a*^2^/(*h*(1 + *q*^2^*a*^2^)), where *q* is the constant for autocatalytic saturation. The inhibition of *a* by *h* is modelled by the *h* term in the denominator. In equation (2.2), the production of inhibitor is proportional to *a*^2^ and degrades at a rate *μ*_h_. In the first two equations, the production of activator and inhibitor is proportional to the cell density *N.* For equation (2.3), we chose a well-known mathematical form [7,15] for the chemotactic term, to reflect the experimentally known fact that BMP-2 serves as a chemo-attractant agent for human vascular smooth muscle cells [19]. Chemotactic migration following the activator gradient is regulated by a coefficient *χ* and saturates at high levels of *a*; *k*_n_ is the constant for this saturation. For cell proliferation, contact inhibition of cell proliferation is a common feature by which cells restrict proliferation and cell division when the culture becomes confluent. It is particularly important for the culture of primary cells such as VMCs [20]. In our experiment, cells reached confluence and stopped proliferating over the first week, and then accomplished the aggregation in 10–14 days. Those considerations lead to the use of logistic growth, where *r*_N_ is the maximum rate of cell proliferation and *N*_c_ is the cell density at confluence.

After non-dimensionalization, the equations may be expressed as:2.4
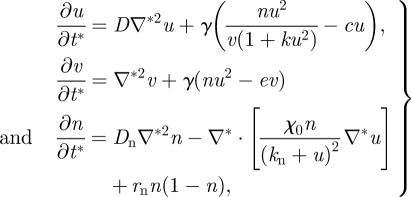
where the dimensionless variables are
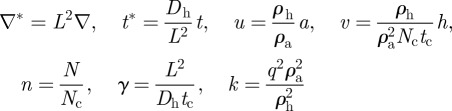

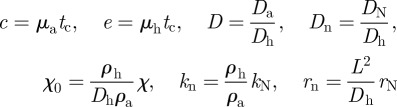
in which *L* is the linear dimension of the domain and *t*_c_ is the characteristic timescale of biosynthetic kinetics.

The PDEs were solved via the finite difference method. The two-dimensional domain was discretized with a uniform mesh (200 × 200). For all simulations, no-flux boundary conditions were used, and initial values of *u* and *v* were uniformly distributed with a 2 per cent random fluctuation, whereas the *n* was uniformly distributed without fluctuation. In our application, the identities of the two morphogens are known: the activator *u* is BMP-2, and the rapidly diffusing inhibitor *v* is MGP [9]. Parameter values were estimated from the biological literature, as described previously with some modifications [9]. For the dynamics of morphogens, the ratio of diffusivity *D*_a_ and *D*_h_ was chosen as 1/200 (*D* = 0.005) to recapture the fast diffusing inhibitor when compared with the activator, based on (i) previously measured diffusion rates of the comparable BMP homologue Decapentaplegic, (ii) previous studies of diffusivity of macromolecules in extracellular matrix, and (iii) their molecular masses [9]. The linear degradation was conservatively estimated as 1 per cent of production rate for BMP-2 and 2 per cent for MGP, which leads to *c* = 0.01 and *e* = 0.02. To account for the smaller dimension of the culture plates in these experiments, we reduced the domain length, *L*, to 1.4 cm. To allow for reduced diffusivity owing to the morphogen transportation in the newly synthesized extracellular matrix, the diffusion coefficient for the inhibitory morphogen, *D*_h_ was estimated as 3 × 10^−8^ cm^2^ s^−1^. Using the approximate timescale of biosynthesis (1800 s), the non-dimensional scaling factor, *γ*, becomes 35 000. Assuming cell diffusion and chemotaxis to be of the same order of magnitude, as suggested in literature [7], we then obtained *χ*_0_ = 0.03. Using *r*_N_ = 0.015 h^−1^ (based on the fact that cell number is tripled after 72 h), leads to *r*_n_ = 322 in dimensionless form. The total time t* = 2 for each simulation. The constant in autocatalysis *k* sets the saturating value at specific values of *v*. However, as the level of *v* (i.e. the concentration of MGP) was not known in the experiments, it is difficult to estimate the value of *k*. As such, the suitable range of *k* is calculated in parameter space (Turing space), in which the mechanism is driven by Turing instability. The mathematical conditions to satisfy the Turing instability are:2.5
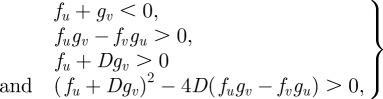
where the subscript denotes the first-order derivative with respect to *u* or *v.* The inequalities are evaluated at the steady state (*u*_0_, *v*_0_). Therefore, the mathematical components in the inequalities can be written as:2.6
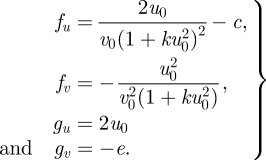


The steady-state value *u*_0_ and *v*_0_ are functions of *k*, *e* and *c*. Given *D* = 0.005, the Turing space can be plotted as function of *k* and *e*/*c* (see also [4]). For *e*/*c* = 2, according to the pre-selected value of *e* and *c*, we can determine the range of *k*, 0 < *k <* 0.34, for which the parameters lie in the linearly unstable region in Turing space. In our simulation, we chose *k* = 0.28.

## 3. Results

### 3.1. Validation of kinetic terms

**Figure 3.**
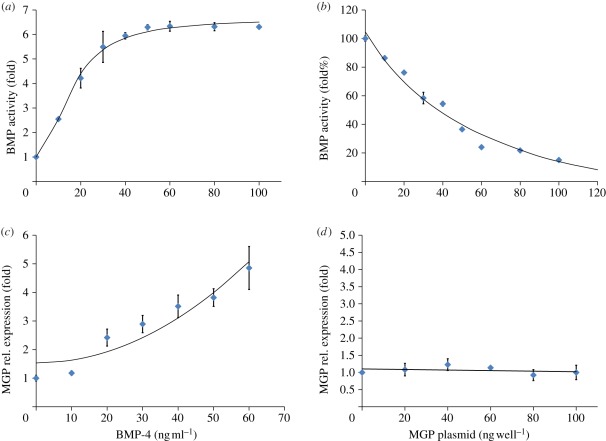
Validation of reaction kinetics. Data are from experiment, while the superimposed curves are functional fits. (*a*) Dependence of increased BMP activity rate on BMP concentration (mean ± s.d.; *n* = 3). Curve is *y* = 76009*x*^2^/(3555568 + 13447*x*^2^) + 1. (*b*) Dependence of BMP activity rate on MGP (mean ± s.d.; *n* = 3). Curve is *y* = 457590/(3040 + 46*x*)−46. (*c*) Dependence of MGP rate on BMP (mean ± s.d.; *n* = 4). Curve is *y* = 0.001*x*^2^ + 1.53. (*d*) Dependence of MGP rate on MGP (mean ± s.d.; *n* = 4). Curve is *y* = −0.0008*x* + 1.1.

To validate the form of the kinetic terms in our mathematical model, we carried out a series of experiments in cells in culture. Experiments validated the critical chemical kinetic terms in our model (figure 3). The increased rate of BMP production as a function of BMP concentration showed good agreement with a *u*^2^/(1 + u^2^) fit (figure 3*a*). The rate of BMP activity was also found to depend negatively on MGP concentration, showing a 1/*v* dependence (figure 3*b*). The MGP production rate was found to increase nonlinearly with BMP, showing decent agreement with the *u*^2^ term in the model (figure 3*c*). Finally, MGP production was shown to have a neutral or even slightly negative dependence on MGP (figure 3*d*). Of note, the model assumes that the rate of MGP production is proportional to *ev*, but *e* is very small (0.02), which is in agreement with our finding.

### 3.2. Control of random cell motility

We controlled random cell motility using the ROCK inhibitor Y27632, applied throughout the culture process. ROCK activates the phosphorylation of NMM-II and inactivates myosin phosphatase [21]. Inhibition of ROCK speeds up cytokinetic migration, presumably through the promotion of the turnover of focal adhesions [22]. We experimentally determined random motility coefficients (*D*_N_) using a wound-healing cell migration assay [17]. As described by Fisher's equation applied to this artificial ‘wound’ within a confluent cell layer, *D*_N_ was derived by calculating the speed of invasion of a cell front, together with the proliferation rate *r*_N_ : *D*_N_ = *V*^2^/4*r*_N_, where *V* is the speed of advance of the cell front and *r*_N_ the maximal proliferation rate [17]. To create artificial wounds in culture, we used shadow mask plating, which creates 300-μm-wide stripes of cells and cell-free regions after plating cells through the mask (figure 1). Thus, *V* can be estimated by the expanding width of the cell stripes, as shown by immunofluorescent staining of NMM-II under different culture conditions (figure 2*a*–*c*). In normal culture conditions (figure 2*a*), analysis yielded a value of *V* = 6.5 ± 1.5 μm h^−1^, leading to *D*_N_ = 2 × 10^−9^ ± 8.8 × 10^−10^ cm^2^ s^−1^ (mean ± s.d.; *n* = 38 images). This result is consistent with the value reported by vascular smooth muscle cells on fibronectin substrate (1.8 × 10^−9^ ± 4 × 10^−10^ cm^2^ s^−1^) [23]. Importantly, we found that in the presence of the ROCK inhibitor (figure 2*b*), *D*_N_ increased approximately ninefold when compared with normal culture conditions (mean ± s.d.: 17.9 × 10^−9^ ± 4.6 × 10^−9^ cm^2^ s^−1^; *n* = 45; figure 2*d*).

### 3.3. Multicellular pattern formation

**Figure 4.**
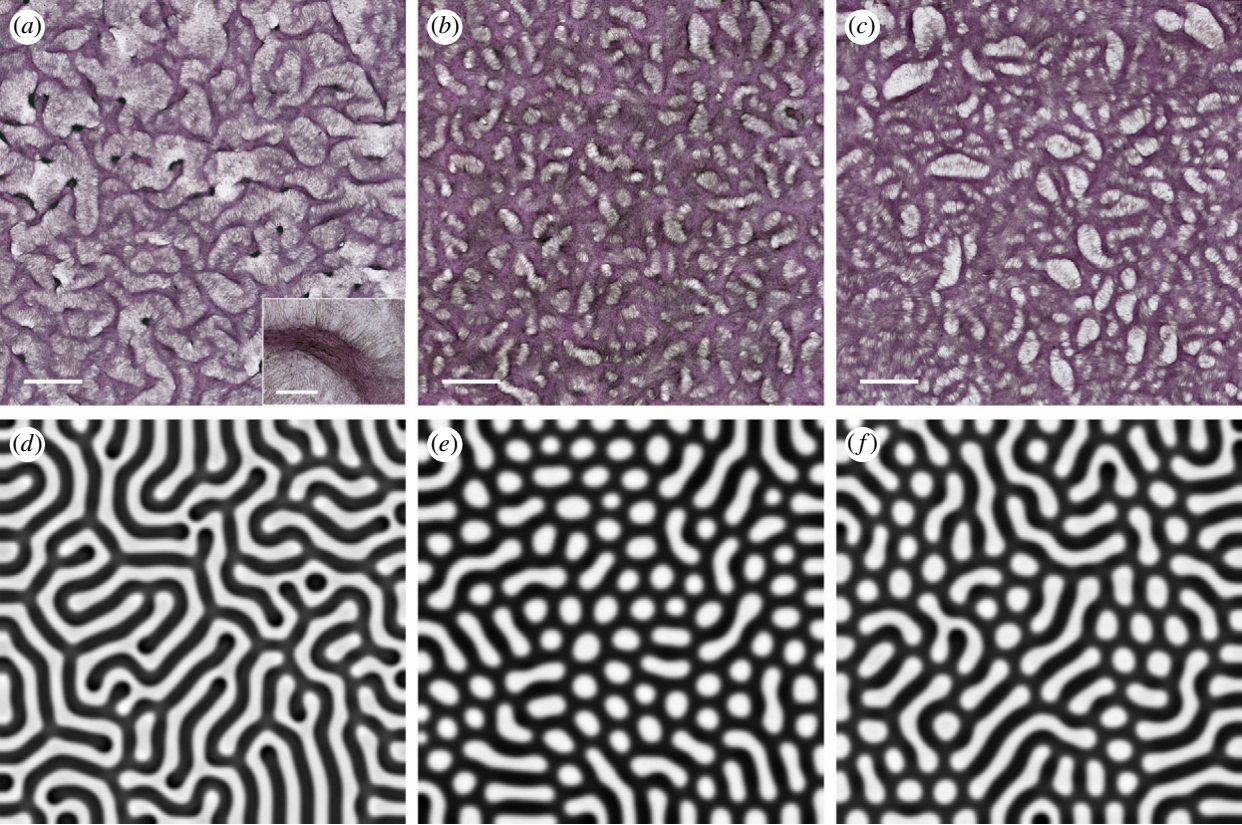
Increased random cell motility alters pattern formation in multicellular aggregates of cultured VMCs from labyrinths to periodic holes. (*a*–*c*) Self-organized VMC pattern in (*a*) normal condition, or with (*b*) ROCK inhibition or (*c*) NMM-II inhibition. Aggregates were stained with haematoxylin. Insets in (*a*), higher magnification of multicellular aggregates. Scale bar, 2 mm and 300 µm (insets). (*d*–*f*) Computational simulations showing *n*(*x*, *y*) as (*d*) a labyrinthine pattern reflecting the normal culture condition (*D*_n_ = 0.06) and periodic holes (*e*) reflecting ROCK inhibition (*D*_n_ = 0.6) and (*f*) NMM-II inhibition (*D*_n_ = 0.2).

After 10–14 days of culture in the presence of the ROCK inhibitor, the VMCs did not form their typical labyrinthine patterns (figure 4*a*), instead forming patterns of periodic holes (figure 4*b*). To exclude other downstream cytokinetic effects of Y27632, we also treated the VMC cultures with blebbistatin, a more specific inhibitor of NMM-II. In the presence of blebbistatin (figure 2*c*), *D*_N_ increased over threefold (mean ± s.d.: 7.3 × 10^−9^ ± 2.6 × 10^−9^ cm^2^ s^−1^; *n* = 37; figure 2*d*), and patterns of holes were again observed (figure 4*c*). These findings suggest that random cell motility affects pattern configuration.

### 3.4. Mathematical modelling reflecting the increase of random cell motility

Numerical simulations using *D*_n_ = 0.06 yielded labyrinthine patterns of *n*(*x*, *y*), resembling the labyrinthine patterns of VMCs in the control cultures (figure 4*d*). In contrast, *D*_n_ = 0.6 or 0.2, as a reflection of increased random motility under ROCK or NMM-II inhibition, yielded periodic hole patterns in *n*(*x*, *y*) (figure 4*e*,*f*), showing consistency with our experiments. As random cell motility tends to move cells away from aggregates, these results suggest that this enhanced dissociation promotes the connectivity between aggregates, ultimately leading to fully connected networks with periodic holes.

## 4. Discussion

Our experiments provide an interesting further application of the Turing paradigm in biology with measurable parameters and experimental controls. The cultures produce a novel pattern for these cells, periodic holes, when perturbed by interventions that increase cell motility, as predicted by our mathematical model. It may therefore be seen as a very elementary model for how to engineer certain forms of tissue by using the cells' own self-organization mechanisms to guide the cells into preferred patterns.

Our mathematical model has limitations, such as the absence of cell–cell interactions. It is becoming increasingly clear that mechanical factors via cell–cell contacts play a significant role in morphogenesis [18,24]. Ultimate morphology as well as cell fate depends on these factors. We assumed that the spatial spread of the invading cells can be modelled as individual cells undergoing a dispersion process [7,17,18], in both our numerical simulations of pattern formation and in the measurement of random cell motility. Thus, mechanical interactions among individual cells, crowding effects, etc., were not considered. Given that our measured value of random cell motility is consistent with the value determined by tracing the migration of individual human vascular smooth muscle cells [23], we suggest that although mechanical interactions among cells may play a role, this factor should be less important in the early stages of pattern formation.

In addition, the inhibition of ROCK and NMM-II may play a role in affecting other intracellular dynamics, such as morphogen production. For example, in cardiomyocytes, a recent report suggested that the expression of BMP-2 is upregulated in the presence of Y27632 [17]. In addition, exogenous BMP-2 may also activate the Wnt-β-catenin and Wnt-planar cell polarity signalling pathways to facilitate vascular smooth muscle motility [25]. For simplicity, those factors were not formulated in the present model. The fact that the ROCK inhibitor and blebbistatin gave similar patterns despite using different cellular pathways suggests that NMM-II inhibition may be the critical factor.

The particular pattern we have studied, periodic holes, may have value as a model for certain kinds of physiological tissue formation. While the terminology ‘periodic holes’ is common, other groups (such as Danino *et al.* [4]) use another appropriate term, ‘inverse spots’. This morphology could be important for pattern formation in physiology. The best physiological example of a structure of periodic holes is trabecular or spongy tissue such as bone. One physiologically critical question is: what determines hole size (relative to the surrounding cellular structure) in such a tissue? That would have important implications for the pathophysiology and potential therapy for osteoporosis, which is an enlargement of the holes in bone. Cortical bone has the smallest holes and thickest network connections, cancellous bone has larger holes and thinner connections, and osteoporotic bone has the largest holes and thinnest connections. Understanding the mechanisms of cellular pattern formation may guide the cell-based therapy on potential treatments for such diseases. Another application is to the formation of the cell wall: Danino *et al.* [4] found a holes regime in three dimensions with uniformly thin ‘sheet-like structures’ that resemble cell walls.

This work attempts to bridge the gap between theoretical analysis and the real biological development by using a cell culture model. The findings may have implications for both normal and pathological development as well as for tissue engineering. Given the importance of treating degenerative diseases, our finding has the potential for clinical translation to reconstitute the morphological features of tissue, to restore, rebuild or improve a functional replacement for injured or otherwise pathological tissue.
